# High Vitamin C Status Is Associated with Elevated Mood in Male Tertiary Students

**DOI:** 10.3390/antiox7070091

**Published:** 2018-07-16

**Authors:** Juliet M. Pullar, Anitra C. Carr, Stephanie M. Bozonet, Margreet C. M. Vissers

**Affiliations:** Centre for Free Radical Research, Department of Pathology and Biomedical Science, University of Otago, Christchurch, P.O. Box 4345, Christchurch 8140, New Zealand; anitra.carr@otago.ac.nz (A.C.C.); stephanie.bozonet@otago.ac.nz (S.M.B.); margreet.vissers@otago.ac.nz (M.C.M.V.)

**Keywords:** vitamin C, ascorbate, plasma, mood, total mood disturbance, POMS

## Abstract

Micronutrient status is thought to impact on psychological mood due to the role of nutrients in brain structure and function. The aim of the current study was to investigate the association of vitamin C status with mood state in a sample of male tertiary students. We measured fasting plasma vitamin C levels as an indicator of vitamin C status, and subjective mood was determined using the Profile of Mood States (POMS) questionnaire. One hundred and thirty-nine male students aged 18 to 35 years were recruited from local tertiary institutes in Christchurch, New Zealand. The average plasma vitamin C concentration was 58.2 ± 18.6 (SD) µmol/L and the average total mood disturbance score was 25.5 ± 26.6 (possible score −32 to 200 measuring low to high mood disturbance, respectively). Plasma vitamin C concentration was inversely correlated with total mood disturbance as assessed by POMS (r = −0.181, *p* < 0.05). Examination of the individual POMS subscales also showed inverse associations of vitamin C status with depression, confusion, and anger. These findings suggest that high vitamin C status may be associated with improved overall mood in young adult males.

## 1. Introduction

Evidence is accumulating that increased consumption of fruit and vegetables is associated with enhanced mood and psychological well-being [1,2,3,4,5]. While it is possible that fruit and vegetable intake is simply a marker of a “healthier” lifestyle, fruit and vegetables are rich in micronutrients, and there are a number of these that may contribute to an effect on mood [6,7]. Fatigue and depression are known to closely precede the physical symptoms of scurvy—a disease caused by vitamin C deficiency [8,9]—suggesting that vitamin C may also be a moderator of mood. Although known for its antioxidant properties, vitamin C (ascorbate) is also a cofactor for a family of biosynthetic and regulatory enzymes with important functions throughout the body. Critically, it is required for the synthesis of the monoamine neurotransmitters dopamine, noradrenaline, and possibly serotonin [10], deficiencies and dysregulation of which have been hypothesised to contribute to depression [11]. Vitamin C is also a cofactor for enzymes involved in the synthesis of carnitine, which is required for the generation of metabolic energy and has been implicated in the fatigue and lethargy associated with scurvy [10,12]. Furthermore, vitamin C regulates the epigenome; it is a cofactor for enzymes involved in both DNA and histone demethylation [13,14]. Epigenetic modifications provide a mechanism by which environmental signals, such as stress, can alter gene expression and neural function and thereby affect behaviour, cognition, and mental health [15].

Vitamin C levels are tightly regulated throughout the body, and its distribution is generally thought to reflect a functional requirement [16]. Concentrations are highest in the brain and other neuroendocrine tissues such as the pituitary and adrenal glands [17]. Indeed, animal models have shown that the brain is the last organ to be depleted of vitamin C during prolonged deficiency, suggesting a vital role in this tissue [18,19].

Several observational studies have suggested a relationship between vitamin C status—typically measured by dietary intake—and mood [20,21,22]. These are further supported by a number of small intervention trials in which participants were supplemented with oral vitamin C [23,24,25,26,27]. Hoffer and co-workers found that supplementation with 1 g/day reduced mood disturbance and psychological distress in acutely hospitalised patients [24]. Similarly, a reduction in anxiety was observed in high school students given 500 mg/day of vitamin C compared to a placebo [23]. We have shown an improvement in subjective mood in a group of male tertiary students supplemented with two gold kiwifruit per day, a food source particularly high in vitamin C (~130 mg vitamin C per kiwifruit). A significant effect was observed in those individuals with higher total mood disturbance at baseline [25]. In addition to a decrease in total mood disturbance, a decrease in fatigue, an increase in vigour, and a trend towards a decrease in depression were demonstrated.

The aim of the current study was to investigate the association of vitamin C status with subjective mood. We measured fasting plasma vitamin C levels as an indicator of vitamin C status in a sample of male tertiary students, and subjective mood was determined using a Profile of Mood States (POMS) questionnaire. This test has been validated and shown to be reliable for assessing mood states [28].

## 2. Materials and Methods

### 2.1. Study Design

This cross-sectional survey was undertaken between April and September 2012. Sample size calculations based on our previous vitamin C studies [29,30] with a standard deviation of 16 µmol/L at 5% type I error indicated 140 participants were required. When sample size was calculated based on the POMS total mood disturbance (TMD) score, a standard deviation of 25 units (as per our previous study [25]) and precision of 5 units at 5% type I error indicated 96 participants were required. A total of 139 male tertiary-level students aged 18 to 35 years and residing in Christchurch, New Zealand at the time of the study were recruited. Recruitment was through verbal or visual/electronic advertisements at local tertiary institutes. The study received ethical approval from the Upper South B Regional Ethics Committee, Christchurch (ethics reference URB/11/12/048). Informed consent was obtained prior to interview and sampling. At the interview, the participants completed a mood questionnaire, a health and lifestyle questionnaire, and blood samples were taken. Participants’ height and weight were recorded by the study interviewer, and the body mass index (BMI) was calculated (kg/m^2^).

### 2.2. Vitamin C Analysis

Fasting peripheral blood was collected by venipuncture into 4 mL K_3_-EDTA vacutainer tubes (Becton Dickinson, Auckland, New Zealand) and immediately placed on ice. Samples were centrifuged at 4 °C to separate plasma, and this was mixed with an equal volume of ice-cold 0.54 mol/L perchloric acid solution containing the metal chelator diethylene-triamine-penta-acetic acid (DTPA) [29]. After centrifugation, the deproteinated plasma samples were stored at −80 °C prior to analysis by HPLC with electrochemical detection, as described previously [29]. Plasma vitamin C concentration is expressed as µmol/L.

### 2.3. Analysis of Mood

The Profile of Mood States (POMS) questionnaire was used to determine the participants’ mood during the previous week. Scores were calculated using a POMS standard scoring grid (Psychological Assessments, Australia). The form comprises 65 mood-related adjectives, which are rated on a 5-point Likert-type scale ranging from 0 (not at all) to 4 (extremely) and then categorised into six mood subscales: tension-anxiety, depression-dejection, anger-hostility, vigour-activity, fatigue-inertia, and confusion-bewilderment. A TMD score is calculated by adding the depression, fatigue, tension, anger, and confusion sub-scores and then subtracting the vigour score. TMD scores range from −32 to 200; a higher score indicates more severe mood disturbance [28].

### 2.4. Statistical Analysis

Data are represented as mean ± SD and 95% confidence intervals. Correlations were tested using Pearson’s Correlation Coefficient with SPSS software (version 22, IBM Corp. Armonk, NY, USA) and differences between nonparametric independent samples used the Mann–Whitney U test; *p* values ≤ 0.05 were considered significant.

## 3. Results

One hundred and thirty-nine male students aged 18 to 35 years were recruited from local tertiary institutes in Christchurch, New Zealand. No exclusion criteria were applied. The baseline characteristics of the study participants are presented in Table 1. The range of plasma levels was 5 µmol/L to 101 µmol/L, with a mean fasting plasma vitamin C concentration of 58 µmol/L. These are normal fasting values. The majority of the cohort had adequate vitamin C concentrations of 50 µmol/L or greater (71%). Roughly one quarter of participants had inadequate vitamin C status of 23–50 µmol/L [31], 2% were marginal (i.e., 11–23 µmol/L), and 0.7% had actual vitamin C deficiency (i.e., <11 µmol/L). The average TMD score of the participants was 25.5, which is similar to values obtained for male college students in the United States [28].

We investigated the relationship between vitamin C status, as assessed by plasma vitamin C concentration, and subjective mood (Table 2). Plasma vitamin C concentration was inversely correlated with total mood disturbance, as assessed by POMS (r = −0.181, *p* < 0.05). Examination of the individual POMS subscales also showed inverse associations of vitamin C status with depression and anger.

**Figure 1:**
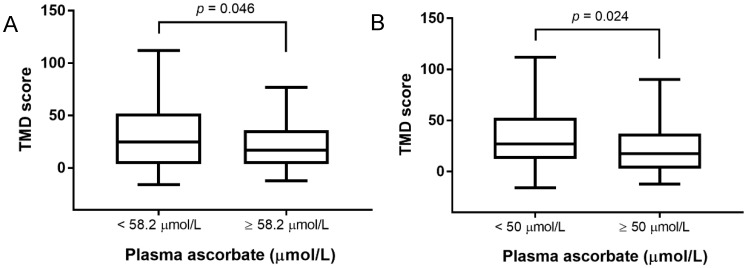
Relationship between total mood disturbance (TMD) score and plasma vitamin C concentration. (**A**) Participants were divided around the mean plasma vitamin C concentration of 58.2 µmol/L. (**B**) Participants were divided around adequacy of vitamin C status (a plasma concentration of 50 µmol/L indicates adequacy). Box plots show median TMD score with the 25th and 75th percentiles as boundaries; whiskers indicate the minimum and maximum of all the data. The TMD score was significantly different between the two groups for each graph (Mann–Whitney U test on ranks).

Furthermore, when participants were split into two groups around either the average plasma vitamin C concentration of 58.2 µmol/L or the adequacy of their vitamin C status (50 µmol/L cut-off), higher total mood disturbance, as assessed by POMS, was associated with lower plasma vitamin C concentration (Figure 1A,B). When participants were divided around the mean plasma vitamin C concentration, median TMD scores were 25 (IQR 4-52) in the low vitamin C group and 17 (IQR 4-36) in the high vitamin C group. Similarly, participants split around the adequacy of vitamin C status had a median TMD score of 27 (IQR 13-53) in the inadequate group and 17.5 (IQR 3-37) in the adequate group. In addition, those with adequate vitamin C status had significantly lower POMS subscores for depression and confusion as compared to those with inadequate status (Table 3).

## 4. Discussion

The brain and central nervous system have a requirement for specific dietary nutrients [32,33]. Supplementation studies have shown an improvement in symptoms for certain mental health disorders with intake of nutrient formulations [34,35,36,37,38]; however, nutrients are also likely to be vital for normal psychological functioning and well-being in healthy individuals. The specific nutrients that are important for brain health are still being investigated. In the present study, we found a significant association between vitamin C status and current mood state in a sample of young adult males. Those individuals with the highest plasma vitamin C concentrations were more likely to have elevated mood.

Mood refers to a positive or negative emotional state of varying intensity that changes in response to life circumstances [39]. Mood is considered long-lasting in contrast to the more acutely experienced emotions. In our study, we used the POMS questionnaire to measure mood state during the previous week. As well as providing a total mood score, POMS gives five different measures of negative mood (depression, fatigue, tension, anger, and confusion) and a single measure of positive mood (vigour). In addition to the relationship observed with overall mood, we have shown significant inverse correlations of vitamin C status with the depression, anger, and confusion subscores in the young men studied. No relationship was observed with the positive mood state vigour despite our previous studies showing an increase in feelings of vigour with a food-based intervention that markedly elevated vitamin C levels [25] and despite emerging evidence for the association of dietary factors with positive well-being [1]. It should be noted that in the study cohort, there were only a few individuals with low vitamin C status of <23 µmol/L, meaning we were unable to investigate the mood state of this group. Rather, our results have shown that those with adequate vitamin C status (>50 µmol/L) tended to have an elevated mood.

One of the best-established functions of vitamin C is in the regulation of neurotransmitter biosynthesis, including that of catecholamines dopamine, norepinephrine, and epinephrine. Vitamin C acts as a cofactor for the enzyme dopamine β-hydroxylase, which converts dopamine to norepinephrine [40]. Animal models of vitamin C deficiency have shown decreased norepinephrine concentrations [41,42,43]. Furthermore, vitamin C can also recycle tetrahydrobiopterin, which is necessary for activation of tyrosine hydroxylase, the rate-limiting enzyme in catecholamine synthesis that synthesizes the dopamine precursor L-3,4-dihydroxyphenylalanine (L-DOPA) [44]. Similarly, tetrahydrobiopterin is a cofactor for tryptophan hydroxylase [45], the initial and rate-limiting enzyme in the synthesis of the neurotransmitter serotonin. There is also evidence emerging that vitamin C is involved in neuronal maturation and functioning [46]. Indeed, brain neurons contain some of the highest levels of vitamin C observed in any mammalian tissue [46]; glial ascorbate concentrations are much lower by comparison.

While the underlying pathophysiology of depression is not yet fully understood, these effects of vitamin C on neurochemistry may provide a mechanism by which it can affect this disorder. An early hypothesis suggested that deficiencies in dopamine, noradrenaline, and serotonin were responsible for major depressive symptoms [11], with some antidepressants elevating levels of these neurotransmitters in the central nervous system. However, it is now apparent that the molecular basis of depression is significantly more complex. Disturbances in dopamine, noradrenaline, and serotonin neurotransmission itself may contribute to the disorder. A more recent hypothesis suggests that low-grade inflammation and immune dysregulation, possibly as a result of psychosocial stressors, may trigger the development and persistence of depression [47]. For example, cytokines are known to induce depressive-type behaviours, and abnormal expressions of proinflammatory cytokines have been shown in patients with depression [48,49,50]. Oxidative stress markers are also elevated in patients with depression [51]. Vitamin C has a number of anti-inflammatory activities as well as being an excellent antioxidant and reducing agent, and it may be able to modulate some of these responses [12,52,53].

A limitation of the current study is that the data is cross-sectional and does not take into account potential confounders of the relationship between vitamin C status and mood, for example, socioeconomic status or other health behaviours. We did not determine the potential impact of any major recent life events that may affect mood in our cohort. Other unmeasured confounders may also have occurred simultaneously in our participants, such as deficiency in another micronutrient or a lower level of physical activity. Thus, we cannot definitively determine whether the relationship between vitamin C status is direct or, as influenced by the confounders above, indirect or parallel. Additionally, it may be that those with better mental health eat more fruits and vegetables causing higher vitamin C status, that is, higher vitamin C status is a consequence of better mood and mental health. In order to provide evidence of a direct relationship between plasma vitamin C status and mood, well-conducted randomized controlled trials are required. This will allow the direction of the relationship to be firmly established and will also allow the effect of any confounders to be eliminated as these should be evenly distributed between the two groups.

Levels of vitamin C in our cohort were generally higher than has been reported in other similar populations [54,55,56]. For example, a recent study in a cohort from Poland showed 7% of participants were marginally deficient in vitamin C [55], while a United States sample found 12–16% were marginally deficient [54]. Dietary information from our cohort indicated that there were a significant number of individuals who regularly used dietary supplements or consumed fruit juice containing vitamin C. It was our estimation that these dietary vitamin C sources contributed significantly to the high mean plasma status of our cohort. Apparent differences between the study populations may also reflect shortcomings in the sample handling and processing used in the studies described above, as inadequate processing can, and commonly does, increase the proportion of samples which are vitamin C deficient [57].

## 5. Conclusions

In conclusion, our findings suggest a possible relationship between vitamin C status and mood state in young adult male students in New Zealand. The current study is cross-sectional and further well-conducted intervention trials are required for proof of causality. There are a number of biological justifications for a positive effect of vitamin C on mood, particularly owing to its role in brain homeostasis and function.

## Figures and Tables

**Table 1:** Characteristics of individuals who completed the study.

Participant Characteristics	Mean ± SD	95% CI	*n* (%)
Age (years)	21.2 ± 2.5	20.8, 21.6	-
Ethnicity	-	-	-
Maori	-	-	13 (9)
NZ European	-	-	106 (76)
Weight (kg)	81.6 ± 15.9	78.9, 84.3	-
Height (cm)	180 ± 7.3	178.8, 181.3	-
BMI (kg/m^2^)	25.1 ± 4.3	24.4, 25.8	-
Vitamin C (µmol/L)	58.2 ± 18.6	55.1, 61.3	-
Adequate	-	-	99 (71)
Inadequate	-	-	36 (26)
Marginal	-	-	3 (2)
Deficient	-	-	1 (0.7)
TMD score	25.5 ± 26.6	21.0, 30.0	-

TMD score was *n* = 138, otherwise data are for *n* = 139. Plasma vitamin C was classified as deficient <11 µmol/L, marginal 11–23 µmol/L, inadequate 23–50 µmol/L, or adequate >50 µmol/L. TMD, total mood disturbance; CI, confidence interval; NZ, New Zealand; BMI, body mass index.

**Table 2:** Pearson linear correlations of plasma vitamin C with mood.

POMS Subscore	r	*p* Value
Total mood disturbance	−0.181	0.034
Depression	−0.192	0.024
Fatigue	−0.061	0.480
Tension	−0.098	0.255
Anger	−0.172	0.044
Vigour	0.100	0.245
Confusion	−0.148	0.084

Total mood disturbance is the sum of the depression, fatigue, tension, anger, and confusion subscores minus the vigour score; *n* = 138.

**Table 3:** Association of plasma vitamin C adequacy with Profile of Mood States (POMS) mood subscales.

POMS Subscore	*p* Value
Total mood disturbance	0.024
Depression	0.012
Fatigue	0.235
Tension	0.195
Anger	0.131
Vigour	0.453
Confusion	0.022

Participants were divided into two groups based on the adequacy of their vitamin C status (50 µmol/L cut-off). Differences in the TMD subscores were tested using the Mann–Whitney U test on ranks.
